# Editorial: Radiotheranostics: From basic research to clinical application

**DOI:** 10.3389/fmed.2023.1171218

**Published:** 2023-03-21

**Authors:** Kamil G. Gareev, Maxim Shevtsov

**Affiliations:** ^1^Department of Micro and Nanoelectronics, Saint Petersburg Electrotechnical University “LETI”, St. Petersburg, Russia; ^2^Laboratory of Biomedical Nanotechnologies, Institute of Cytology of the RAS, St. Petersburg, Russia; ^3^Personalized Medicine Centre, Almazov National Medical Research Centre, St. Petersburg, Russia; ^4^Institute of Life Sciences and Biomedicine, Far Eastern Federal University, Vladivostok, Russia

**Keywords:** radiotheranostics, precision medicine, nuclear medicine, radiography, nanoparticles

In the last few years, the term “radiothera(g)nostics” (RT) became commonly used in different fields, including theranostics, nanotheranostics, magnetotheranostics, optotheranostics, and phototheranostics (1). In RT, radioactive material (radioisotopes) coupled to targeting vectors are used for therapy and diagnosis of malignant diseases including cancer (2, 3) (Pallares and Abergel). RT often implies positron emission tomography (PET) and radiotherapy for radio-targeted treatments based on the imaging of a target area and concomitantly exposing this area to therapeutic interventions (4). Since the imaging and therapeutic probes have similar chemical properties, both can present equal pharmacokinetics (3). Therefore, the absorbed doses for the therapeutic probe into the tumor and normal tissue can be calculated from the quantitative imaging data provided by PET and single-photon emission computed tomography (5).

RT agents can be labeled with the targeting biomolecules, including small molecules, peptides, and antibodies, and can also contain targeting core materials (e.g., superparamagnetic iron oxide core). One of the most frequently used model peptides, the RGD peptide, can be used as a label for ^211^At radioisotope for cancer RT to provide a highly selective concentration in the tumor tissue (5). The albumin-binding prostate-specific membrane antigen radioligand based on actinium-225 can be promising for targeted α-therapy (6).

RT combined with chemotherapy can improve survival and the quality of life for patients with aggressive anaplastic thyroid cancer. Zhou et al. (7) reported on the tumor-killing effects of ^64^Cu-labeled NPs for RT and combined RT/Near-infrared laser-induced photothermal therapy mediated by a single-compartment nanoparticle platform. In this Research Topic, ^131^I labeled albumin indocyanine green nanoparticles (size 25–45 nm) with near IR light were successfully used for a radio-photothermal ablation of refractory thyroid cancer and SPECT images in mice (Zhang et al.).

RT can also treat non-malignant disorders such as hypertrophic cardiomyopathy in patients who cannot undergo invasive surgery due to pathophysiological conditions. In a case report, a 71-year-old patient was treated with ablative stereotactic radiotherapy (Xiao et al.). The thickness of the septum was drastically reduced from 24 to 19 mm 3 months after therapy as shown by MR imaging of T1 mapping. Stereotactic body radiotherapy in a single session to the heart may offer an alternative for endocardial or epicardial ablation techniques, as it overcomes the general limitation of energy deposition problems and has been already applied for the treatment of a patient with dilated cardiomyopathy (8).

The theranostic pair consisting of ^64^Cu and ^67^Cu has great potential for preparing metal chelates for medical use. Lee et al. describe the production method of ^64^Cu/^67^Cu radioisotopes with the 30 MeV RFT cyclotron. The radioisotope ^64^Cu acts as a nuclide for PET imaging and ^67^Cu serves as a beta emitter for therapy of the same target structure.

The review of Pallares and Abergel describes radiopharmaceuticals approved in the USA and Europe for a targeted α-therapy of non-Hodgkin lymphomas and PSAM-positive prostate carcinoma and outlines the great potential of these targeted therapeutic concepts.

In conclusion, we believe that novel RT techniques and RT agents will be translated into clinical practice in the near future. The further development of RT depends on technological improvements in the synthesis of RT agents and on the creation and implementation of newer medical equipment.

## Author contributions

Both authors listed have made a substantial, direct, and intellectual contribution to the work and approved it for publication.
